# Supplementary material for: Annotation-efficient deep learning for automatic medical image segmentation
Source: Nat Commun. 2021 Oct 8;12:5915. doi: 10.1038/s41467-021-26216-9 (PMC8501087; doi:10.1038/s41467-021-26216-9)
Supplement: Supplementary file 1 — Supplementary Information [file 41467_2021_26216_MOESM1_ESM.pdf]

Supplementary information

Annotation-efficient deep learning for automatic medical image segmentation

Shanshan Wang[1,2,3,11,12]*, Cheng Li[1,11,12]*, Rongpin Wang[4,11], Zaiyi Liu[5], Meiyun Wang[6], Hongna Tan[6], Yaping Wu[6], Xinfeng Liu[4], Hui Sun[1], Rui Yang[7], Xin Liu[1], Jie Chen[2,8], Huihui Zhou[9], Ismail Ben Ayed[10], Hairong Zheng[1,12]*

[1]Paul C. Lauterbur Research Center for Biomedical Imaging, Shenzhen Institutes of Advanced Technology, Chinese Academy of Sciences, Shenzhen, Guangdong, China.

[2]Peng Cheng Laboratory, Shenzhen, Guangdong, China.

[3]Pazhou Laboratory, Guangzhou, Guangdong, China.

[4]Department of Medical Imaging, Guizhou Provincial People's Hospital, Guiyang, Guizhou, China.

[5]Department of Medical Imaging, Guangdong General Hospital, Guangdong Academy of Medical Sciences, Guangzhou, Guangdong, China.

[6]Department of Medical Imaging, Henan Provincial People's Hospital & the People's Hospital of Zhengzhou University, Zhengzhou, Henan, China.

[7]Department of Urology, Renmin Hospital of Wuhan University, Wuhan, Hubei, China.

[8]School of Electronic and Computer Engineering, Shenzhen Graduate School, Peking University, Shenzhen, Guangdong, China.

[9]Brain Cognition and Brain Disease Institute, Shenzhen Institutes of Advanced Technology, Chinese Academy of Sciences, Shenzhen, Guangdong, China.

[10]ETS Montreal, Montreal, Canada.

[11]These authors contributed equally: Shanshan Wang, Cheng Li, and Rongpin Wang.

[12]The corresponding authors are: Hairong Zheng, Shanshan Wang, and Cheng Li.

*****e-mail: hr.zheng@siat.ac.cn; ss.wang@siat.ac.cn; cheng.li6@siat.ac.cn

Supplementary methods

Network architecture

Except for the last convolution with a kernel size of 1×1, all convolutions use 3x3 kernels by default with zero padding of one to keep the spatial resolution. Suppose the input image has a dimension of $\mathrm{H} \times \mathrm{W}$. After the four maxpooling operations (2×2 kernels with a stride of 2), the image dimensions change to $\mathrm{H}/2 \times \mathrm{W}/2$, $\mathrm{H}/4 \times \mathrm{W}/4$, $\mathrm{H}/8 \times \mathrm{W}/8$, and $\mathrm{H}/16 \times \mathrm{W}/16$, respectively, which are gradually recovered to $\mathrm{H} \times \mathrm{W}$ by the upsampling layers. For single-modal inputs, the feature numbers of the five encoder blocks are 64, 128, 256, 512, and 1024. For multi-modal inputs, the extracted feature numbers are divided equally among the multiple modalities so that after the feature fusion, the feature numbers are the same as those extracted from the single-modal inputs. Bilinear upsampling is utilized for our experiments.

CHAOS dataset – segmentation with severely limited annotations

From the 20 cases with high-quality annotations, 10 cases are randomly selected as the test set. The remaining 10 cases and the 20 unannotated cases are utilized as the training set. To simulate the extremely scarce annotation condition, our proposed framework AIDE is evaluated when only 1 high-quality annotated case (30 image samples) is available and the left 29 cases (954 image samples) are used without annotations. Specifically, we train a segmentation network with 1 randomly selected annotated case for 100 epochs. The trained network is then employed to generate pseudo-labels with low qualities for the other 29 cases. With this constructed training set of 30 cases (30 image samples with high-quality annotations and 954 image samples with pretrained model-generated low-quality labels), AIDE is implemented and the segmentation performance on the test set is compared with baseline models trained with the only annotated cases, trained with 10 high-quality annotated cases, and trained with the constructed training set but without the proposed self-label correcting capability. Pseudo-label[1] and co-teaching[2] are adopted as two methods for comparison as well.

Prostate datasets – segmentation with no target domain annotations

We treat each domain as the source domain and test the segmentation performance on the other two domains. For example, when Domain 1 is treated as the source domain with high-quality annotations, models are trained with Domain 1 training dataset. Domain 2 and Domain 3 are regarded as two separate target domains, and the high-quality annotations of the respective training sets are not utilized during network training. Under this setting, we investigate the performance of AIDE when only source domain annotations are available. Similar to our experiments for SSL with the CHAOS dataset, models trained with the source domain annotated training data are applied to generate pseudo-labels for the target domain training data, and these pseudo-labels can be very noisy depending on the discrepancies between the different domains. AIDE is utilized to handle the combined data made of source domain high-quality annotated data and the target domain noisily labeled data to enhance the model performance on the target domain test set. Baseline models include those trained with the respective high-quality annotated data and the combined dataset, as well as pseudo-label[1] and co-teaching[2].

QUBIQ dataset – segmentation with noisy annotations

Respective sets of annotations from different annotators are utilized to train the model. Correspondingly, we treat each individual set of annotations as low-quality annotations and

investigate the effectiveness of AIDE on handling these data. The models trained with noisy annotations are utilized to initialize the network training of AIDE. Multiple annotations are also provided for the test data. Segmentation performance is calculated in an elegant manner. First, the continuous ground-truth annotations are generated by averaging the different annotations. Then, a series of thresholds (0.1, 0.2, 0.3, 0.4, 0.5, 0.6, 0.7, 0.8, and 0.9) are defined to obtain the binary growth truth and prediction segmentation maps. DSCs are calculated at these thresholds and the averaged DSCs are reported.

Evaluations with the clinical breast tumor segmentation datasets

To evaluate the impacts of AIDE on extricating radiologists from the cumbersome image annotation work required by conventional DNNs, we train DNNs with only 10% annotations and compare the segmentation performance with that achieved by training with 100% annotations available and that provided by independent annotators. To confirm that our achievements are robust and generalizable, we conduct independent experiments with the three datasets collected from the three medical centers and report the respective results.

Implementation details of AIDE

Our model was implemented using PyTorch and trained on a Tesla V100 32GB GPU with a batch size of 4. ADAM was utilized as the optimization method. The initial learning rates were set to 0.0001 for all experiments except for the experiments with AIDE on the QUBIQ dataset, for which the initial learning rates were set to 0.00001. Step decay learning rate policy was applied that the learning rates were decayed by 50% after every 30 epochs. During network training, the images are resized to 256 x 256 for the CHAOS data, 384 x 384 for the prostate data, 256 x 256 for the brain growth and brain tumor data, 512 x 512 for the kidney data, and 384 x 384 for the breast tumor data after considering the original image sizes and the computational resources. We train the networks for 100 epochs and report the results on the test set of the model achieving the highest training set DSC during network optimization.

Supplementary results

Influence of labeled data on fully-supervised model's performance

Different numbers of training data are used to investigate the sensitivity of fully-supervised models to training conditions (Supplementary Fig. 1 and Supplementary Table 1). As depicted in Supplementary Fig. 1 (without the post-processing step), more training samples lead to clearly better segmentation results. The enhancement from 1 labeled training case (30 training samples) to 10 labeled training cases (331 training samples) is substantial (17.8% absolute DSC value increase and 25.3% relative increase), which indicates that for conventional fully-supervised deep learning methods, a large dataset with high-quality annotations is required to achieve the expected performance.

Experiments with different levels of noisy labels on the CHAOS dataset

We investigate the influence of the number of noisy labels on segmentation performance systematically and comprehensively with SSL, for which low-quality labels (i.e. noisy labels) are generated by models trained with the limited annotated data. The combined training data (high-quality labeled data and noisily labeled data) are then utilized for network optimization. Results achieved under different experimental conditions are listed in Supplementary Table 1. For the fully-supervised learning baseline, noisy labels already affect their performance when the noise level is over 20% (P = 0.0133 from a paired t-test between the DSCs of Exp. 6 and Exp. 7 in Supplementary Table 1). Compared to the baseline, our method consistently improves the performance (characterized by increased DSC and decreased RAVD) at different noise levels. Furthermore, promising performance is achieved by our method with 97% noisy labels (Exp. 21 in Table Supplementary Table 1). Besides, comparing Exp. 21 to Exp. 19 in Supplementary Table 1, with the same quantity of high-quality labeled data, the introduction of more low-quality labeled data results in improved segmentation performance of our system.

Results also indicate that when a large labeled dataset is provided, AIDE can perform on par to traditional supervised learning. Supplementary Table 1 shows that when all the labels are high-quality labels (Exp. 6 for traditional supervised learning method and Exp. 14 for our proposed method), AIDE can still improve the model performance slightly. If additional noisily labeled training data are provided with this large high-quality labeled data, better results can be achieved (comparing Exp. 20 to Exp. 14).

Ablation studies for different hyper-parameters on the CHAOS dataset

With the proposed framework, several hyper-parameters are introduced, including the temperature, the loss weight, and the warm-up epoch number. We conduct ablation studies to investigate the influence of these hyper-parameters on the model performance (Supplementary Table 2). Results indicate that our method is very robust to the parameters within the respective tested ranges.

Effects of the employed post-processing step

Supplementary Fig. 2 presents several results with or without the post-processing step under different experimental conditions. It can be observed that for low-performance models (e.g. S01 for the fully-supervised baseline without the post-processing step and S02 with the post-processing), the post-processing can sometimes be very important and many outliers can be

removed. Nevertheless, for our proposed method (S11 without the post-processing and S12 with the post-processing), the performance enhancement brought by the post-processing step is marginal.

Baselines for unsupervised domain adaptation

Additional experiments are conducted by model training with both labeled source domain training data and labeled target domain training data (Supplementary Table 3). It can be observed that in most situations, utilizing labeled data from other domains can help achieve better segmentation results (e.g. compare the experiment training with Domain 1 labeled & 2 labeled data and testing on Domain 2 (DSC=89.0%, RAVD=9.19%, ASSD=1.21 mm, and MSSD=7.27 mm) in Supplementary Table 3 to the experiment training with only Domain 2 labeled data and testing on Domain 2 (DSC=87.3%, RAVD=16.1%, ASSD=1.36 mm, and MSSD=7.89 mm) in Table 2 in the manuscript). However, for the baseline comparison methods, when the introduced data from a different domain is unlabeled and pseudo-labels are utilized, the performance on both domains are affected, especially for the data from the domain without training annotations (e.g. the experiment training on Domain 1 labeled and 2 unlabeled data and testing on Domain 2 (DSC=33.7%, RAVD=71.5%, ASSD=5.94 mm, and MSSD=20.68 mm) in Supplementary Table 3). Compared to theses baselines, the performance of our AIDE on the target domain without training data annotations is largely improved (e.g. the experiment training on Domain 1 labeled and 2 unlabeled data and testing on Domain 2 with AIDE (DSC=80.0%, RAVD=25.1%, ASSD=2.83 mm, and MSSD=18.2 mm)).

Visual segmentation maps of the prostate

Supplementary Figs. 3–5 show the example test results when transferring models from Domain 1 to Domain 2, from Domain 1 to Domain 3, and from Domain 2 to Domain 3. These three cases are plotted as big enhancements in the segmentation results of AIDE are observed. Visual results confirm the quantitative characteristics that direct cross-domain model testing is not feasible, and AIDE can help increase the cross-domain model testing accuracy without utilizing any target domain annotations.

Results of methods for comparison on the unsupervised domain adaptation task

Experiments have been conducted to check the performance of the two literature-reported methods (pseudo-label and co-teaching) on the unsupervised domain adaptation task (Supplementary Table 4). Results indicate that the performance is improved when compared to the conventional fully-supervised learning approach with pseudo-labels of the target domain data generated by the pretrained models. Overall, our method achieves better results than these two methods for comparison.

Performance distributions over different thresholds on the QUBIQ datasets

Supplementary Figs. 6 and 7 show DSC values of models tested with different thresholds of the four tasks. It can be summarized that in most cases, AIDE achieves higher DSCs, especially for smaller threshold values, which indicates that AIDE is more robust and less dependent on this threshold parameter.

Visual segmentation maps of breast tumors

Supplementary Figs. 8–10 show the example test results on the three breast datasets, respectively. Overall, both the model predictions and the independent radiologist's manual annotations are very close to the reference labels. Meanwhile, there are a few cases when the manual annotations are more accurate (second rows of each figure). Similarly, there exist cases when the model predictions are better (third rows of each figure). Both the model and the radiologist can have relatively large discrepancies from the reference labels in a few cases (fourth rows of each figure).

The rationale for the proposed label filtering and correction steps

Empirically, we find that when the training data containing noisy annotations, there is an overall positive correlation between the DSCs calculated comparing network outputs to noisy labels and the DSCs calculated comparing noisy labels to the high-quality labels for the training set in the initial 10 epochs (Supplementary Fig. S11a). In other words, within the considered training period, the network cannot memorize well those samples that contain large label noise but can learn the patterns of the samples that contain low label noise. We also check the model performance in the last 10 epochs and find that all the samples are well memorized (Fig. R1b). This phenomenon is relevant to the network memorization pattern, and it has been frequently observed for natural image analysis[3–5]. Here we show that the same network memorization pattern exists for medical image segmentation. Therefore, we can exploit this network property and conduct the label filtering and correction properly to make full use of both the high-quality labels and low-quality labeled image data. Accordingly, we design the specific noisy label updating schedule – we update the suspected noisy labels if the training epoch number is smaller than the defined warm-up epoch number and every 10 epochs thereafter. The first criterion is raised according to the above-mentioned network memorization behavior we observe. The second criterion is raised because after certain epochs, the performances of the networks become relatively stable, and there is no need to update the labels so frequently. Therefore, in our experiments, we update the labels every 10 training epochs.

References

1. Lee, D.-H. Pseudo-label: The simple and efficient semi-supervised learning method for deep neural networks. *ICML 2013 Work. Challenges Represent. Learn.* 1–6 (2013).
2. Han, B. *et al.* Co-teaching: Robust training of deep neural networks with extremely noisy labels. in *Conference on Neural Information Processing Systems (NeurIPS)* (2018).
3. Arplt, D. *et al.* A closer look at memorization in deep networks. in *International Conference on Machine Learning (ICML)* (2017).
4. Jiang, L., Zhou, Z., Leung, T., Li, L.-J. & Li, F.-F. MentorNet: Learning data-driven curriculum for very deep neural networks on corrupted labels. in *International Conference on Machine Learning (ICML)* (2018).
5. Chen, P., Liao, B., Chen, G. & Zhang, S. Understanding and utilizing deep neural networks trained with noisy labels. in *International Conference on Machine Learning (ICML)* (2019).

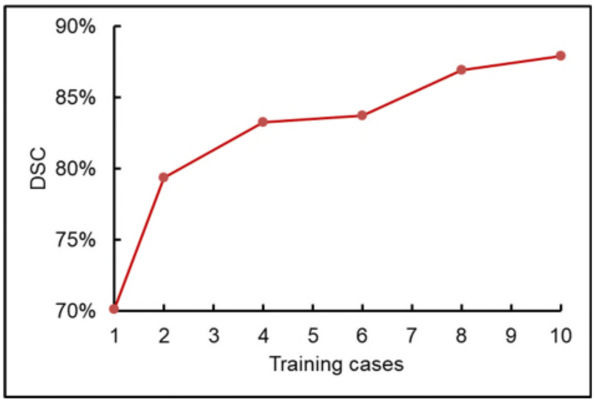

Supplementary Fig. 1 | Network performance on the test set (characterized by Dice score) with 1, 2, 4, 6, 8, or 10 training cases with high-quality labels of the CHAOS dataset. More training samples lead to improved segmentation performance.

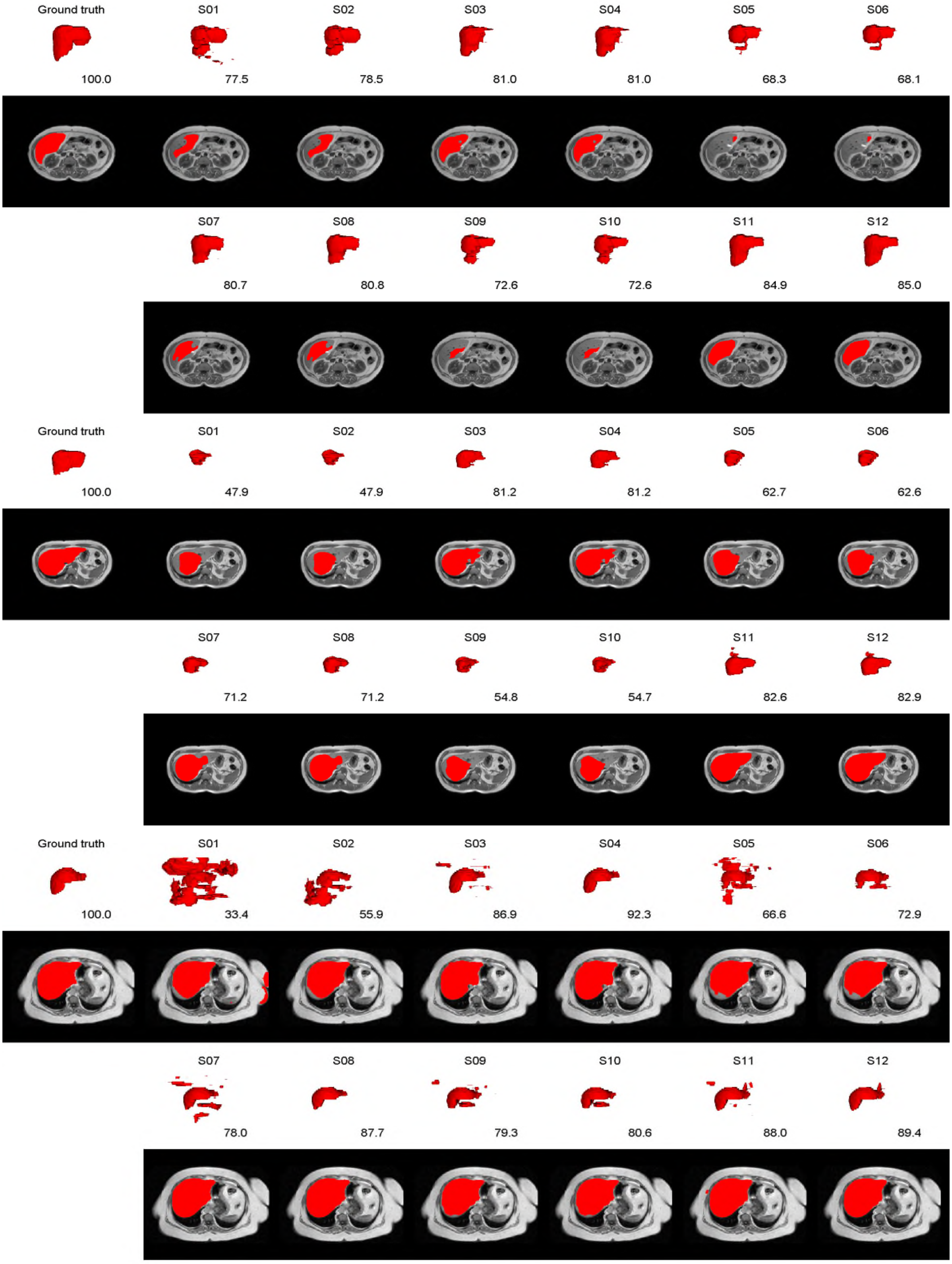

Supplementary Fig. 2 | Visualization of segmentation results for SSL on the CHAOS dataset. S01 to S12 refer to different experimental settings in Table 1. Numbers are the DSC values (%).

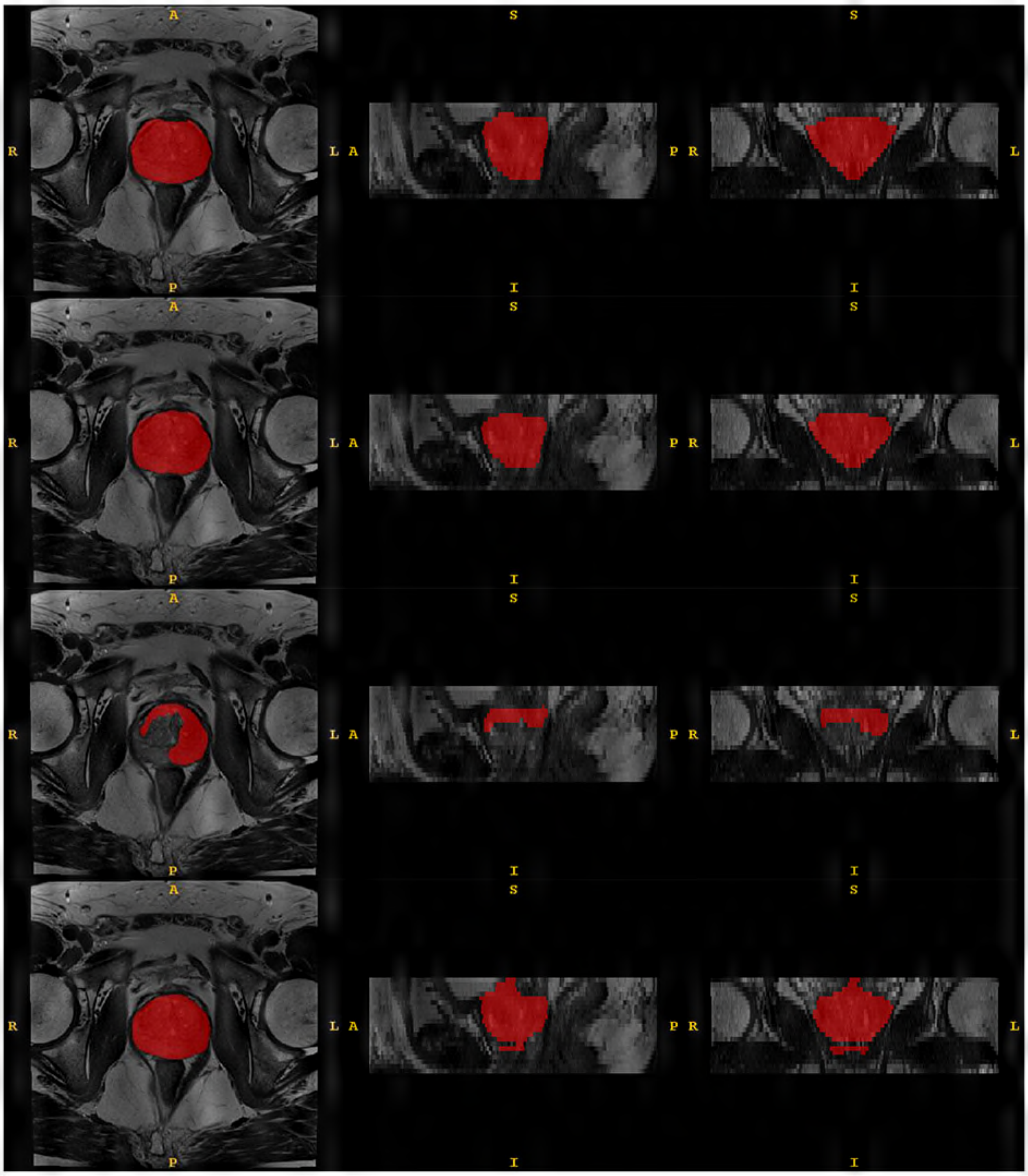

Supplementary Fig. 3 | Prostate segmentation maps of one case when transferring models from Domain 1 dataset to Domain 2 dataset. From left to right, the three columns correspond to the transverse plane image, the sagittal plane image, and the coronal plane image. From the head to bottom, the four rows refer to the ground-truth manual segmentation, the outputs of models trained directly on Domain 2 training set, the outputs of models trained on Domain 1 training set, and the outputs of models trained on Domain 1 training set with manual annotations and Domain 2 training set without annotations with AIDE.

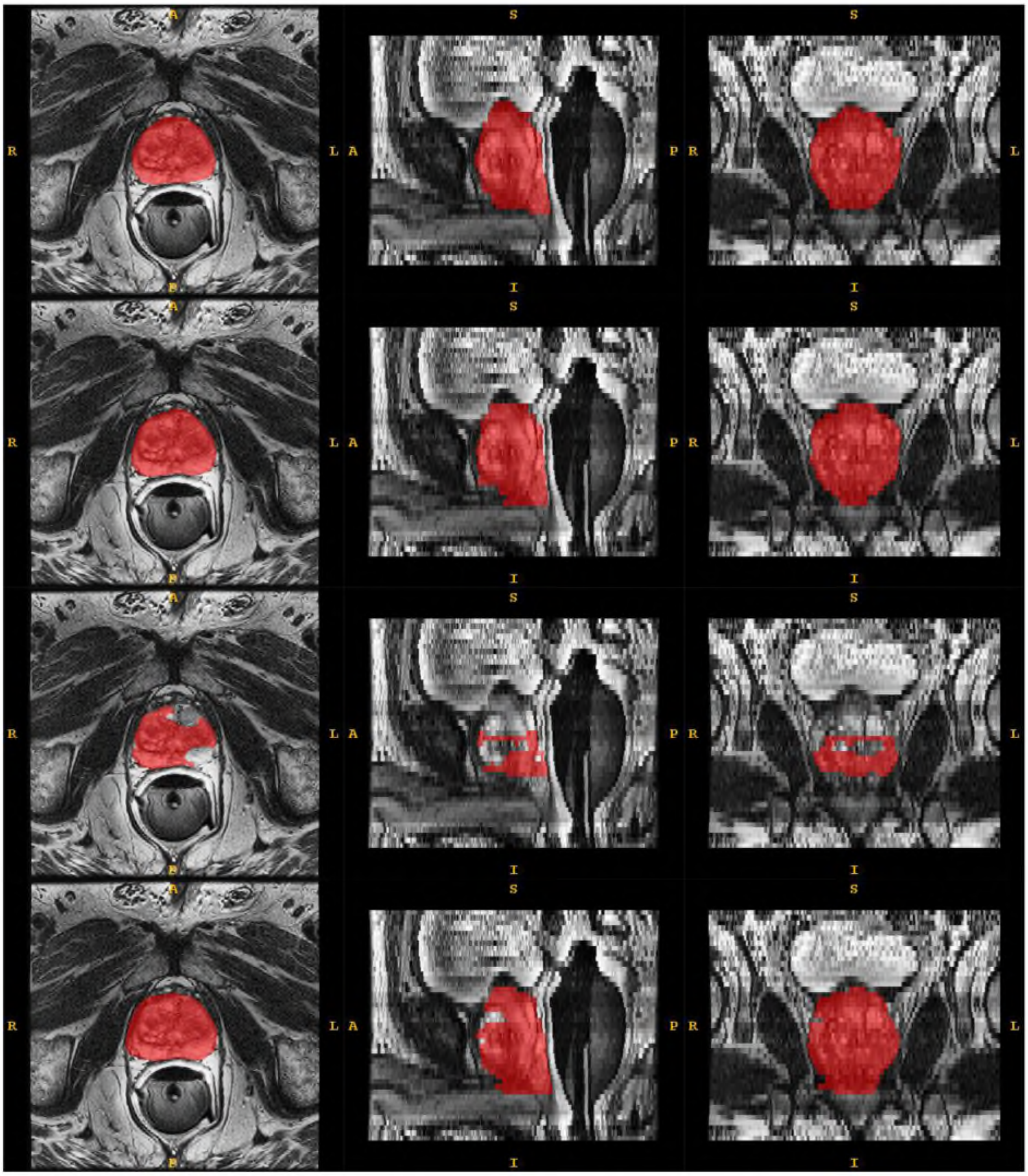

Supplementary Fig. 4 | Prostate segmentation maps of one case when transferring models from Domain 1 dataset to Domain 3 dataset. From left to right, the three columns correspond to the transverse plane image, the sagittal plane image, and the coronal plane image. From the head to bottom, the four rows refer to the ground-truth manual segmentation, the outputs of models trained directly on Domain 3 training set, the outputs of models trained on Domain 1 training set, and the outputs of models trained on Domain 1 training set with manual annotations and Domain 3 training set without annotations with AIDE.

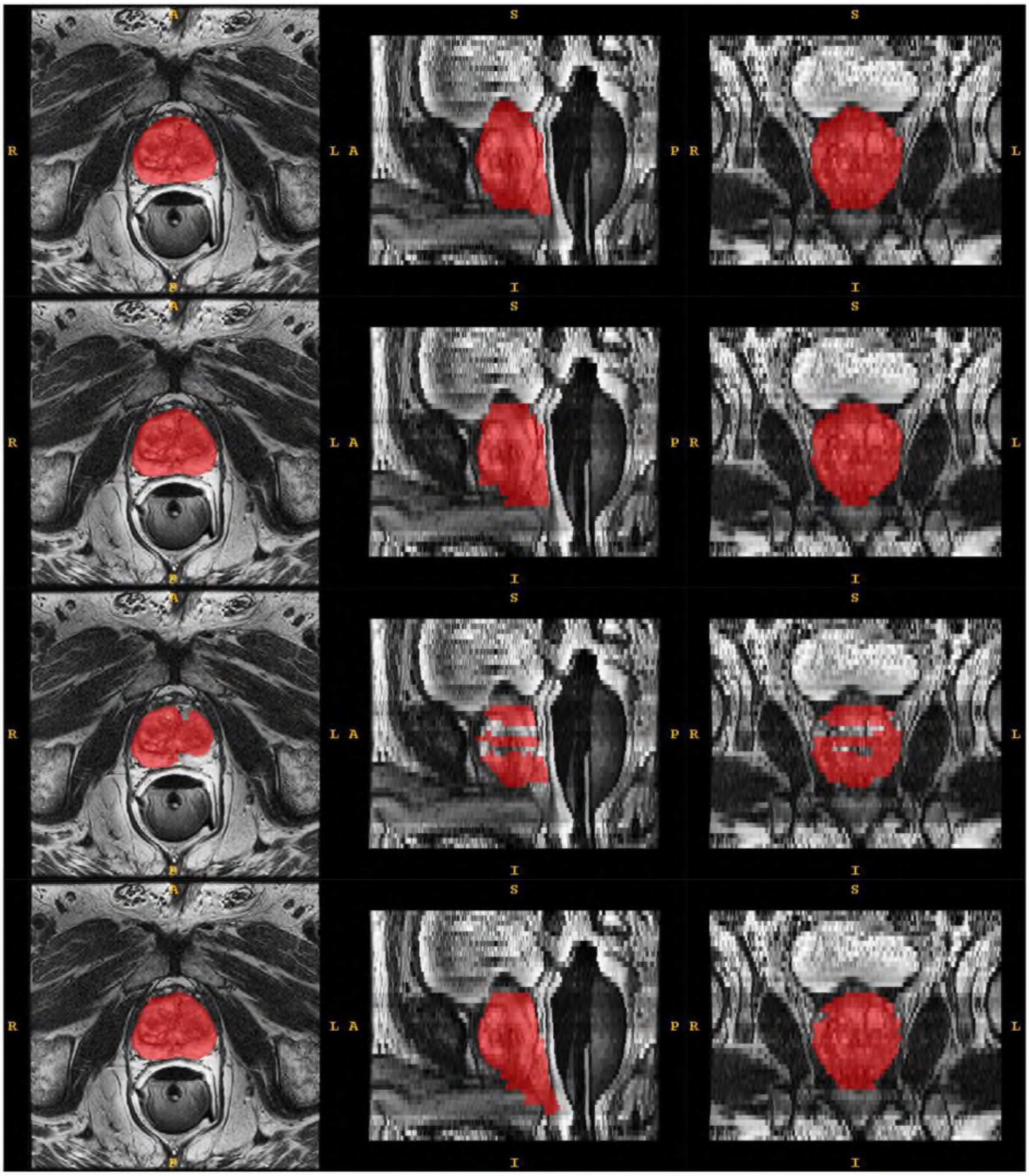

Supplementary Fig. 5 | Prostate segmentation maps of one case when transferring models from Domain 2 dataset to Domain 3 dataset. From left to right, the three columns correspond to the transverse plane image, the sagittal plane image, and the coronal plane image. From the head to bottom, the four rows refer to the ground-truth manual segmentation, the outputs of models trained directly on Domain 3 training set, the outputs of models trained on Domain 2 training set, and the outputs of models trained on Domain 2 training set with manual annotations and Domain 3 training set without annotations with AIDE.

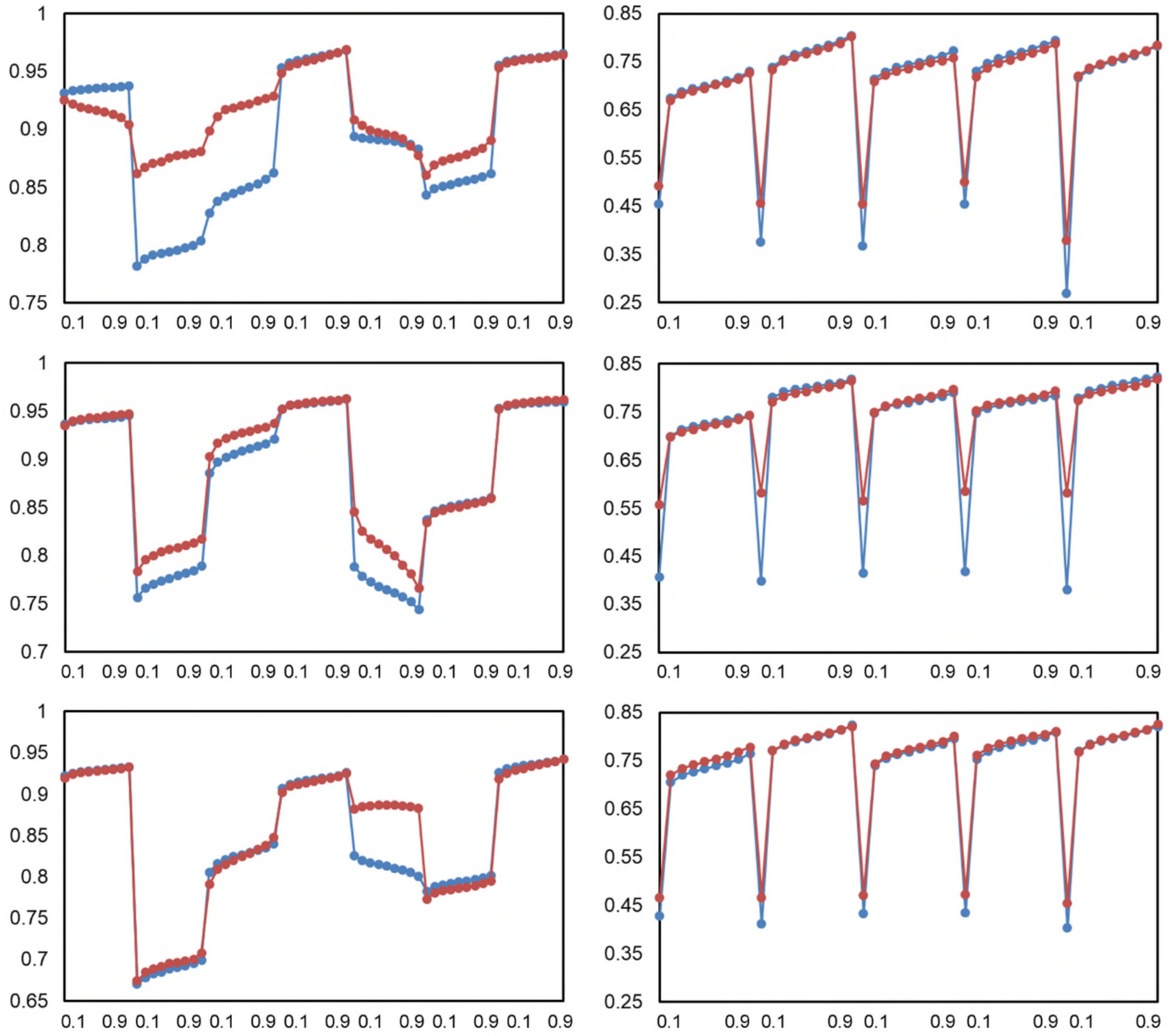

Supplementary Fig. 6 | Segmentation results of the QUBIQ dataset Task1 and Task2. Vertical axis is DSC. Horizontal axis indicates the threshold utilized to calculate the DSC. The left three figures correspond to the results on Task1 with Anno1, Anno2, and Anno3 as the training annotations, respectively. The right three figures show the results on Task2 with Anno1, Anno2, and Anno3 as the training annotations. Blue lines are the results obtained by conventional models and red lines are the results obtained with AIDE.

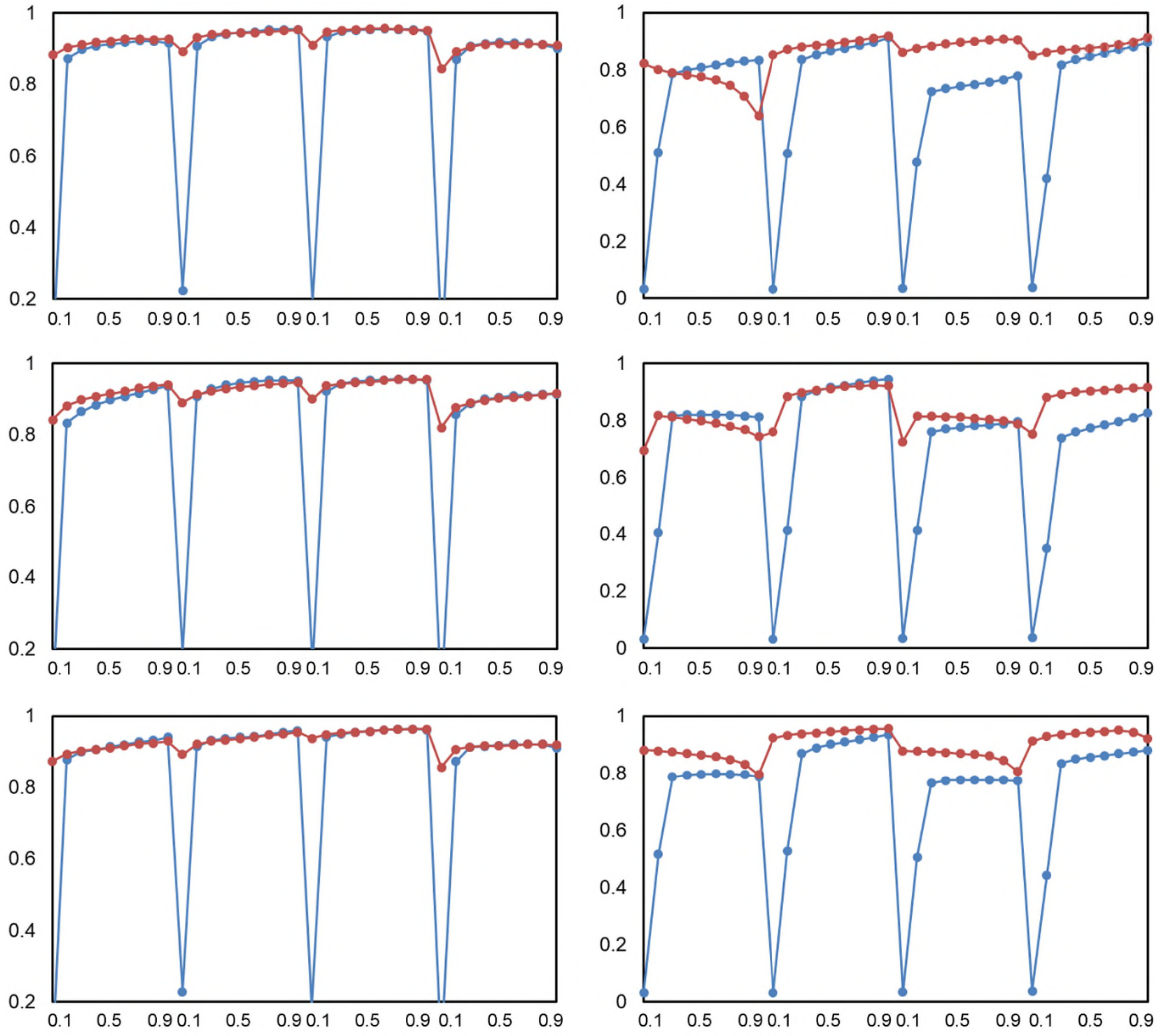

Supplementary Fig. 7 | Segmentation results of the QUBIQ dataset Task3 and Task4. Vertical axis is DSC. Horizontal axis indicates the threshold utilized to calculate the DSC. The left three figures correspond to the results on Task3 with Anno1, Anno2, and Anno3 as the training annotations, respectively. The right three figures show the results on Task4 with Anno1, Anno2, and Anno3 as the training annotations. Blue lines are the results obtained by conventional models and red lines are the results obtained with AIDE.

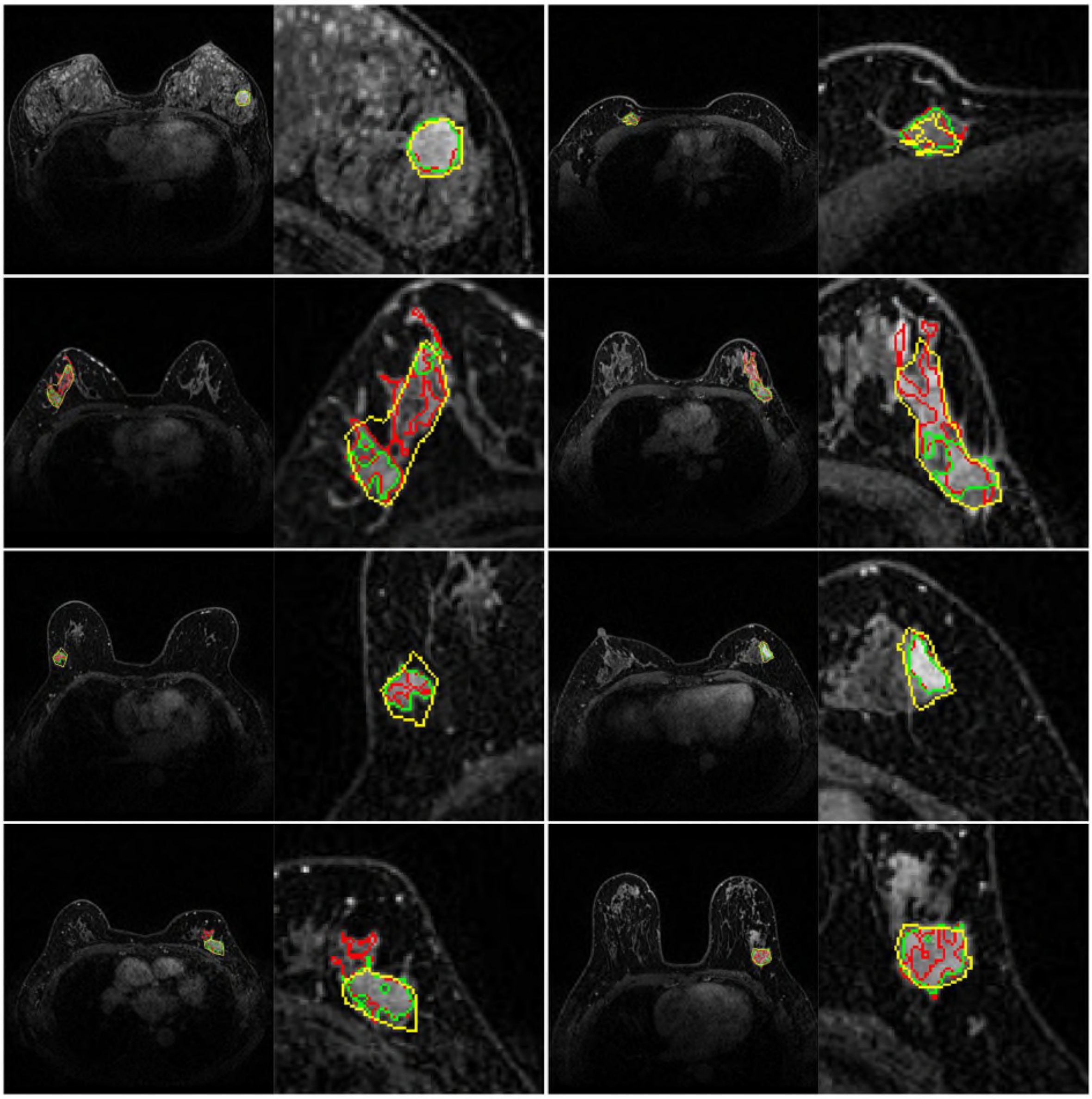

Supplementary Fig. 8 | Example breast tumor segmentation on the GGH dataset. Red lines indicate the boundaries of the high-quality annotations. Green lines are the segmentation results of AIDE with 10% training annotations. Yellow lines refer to the segmentations obtained from an independent radiologist.

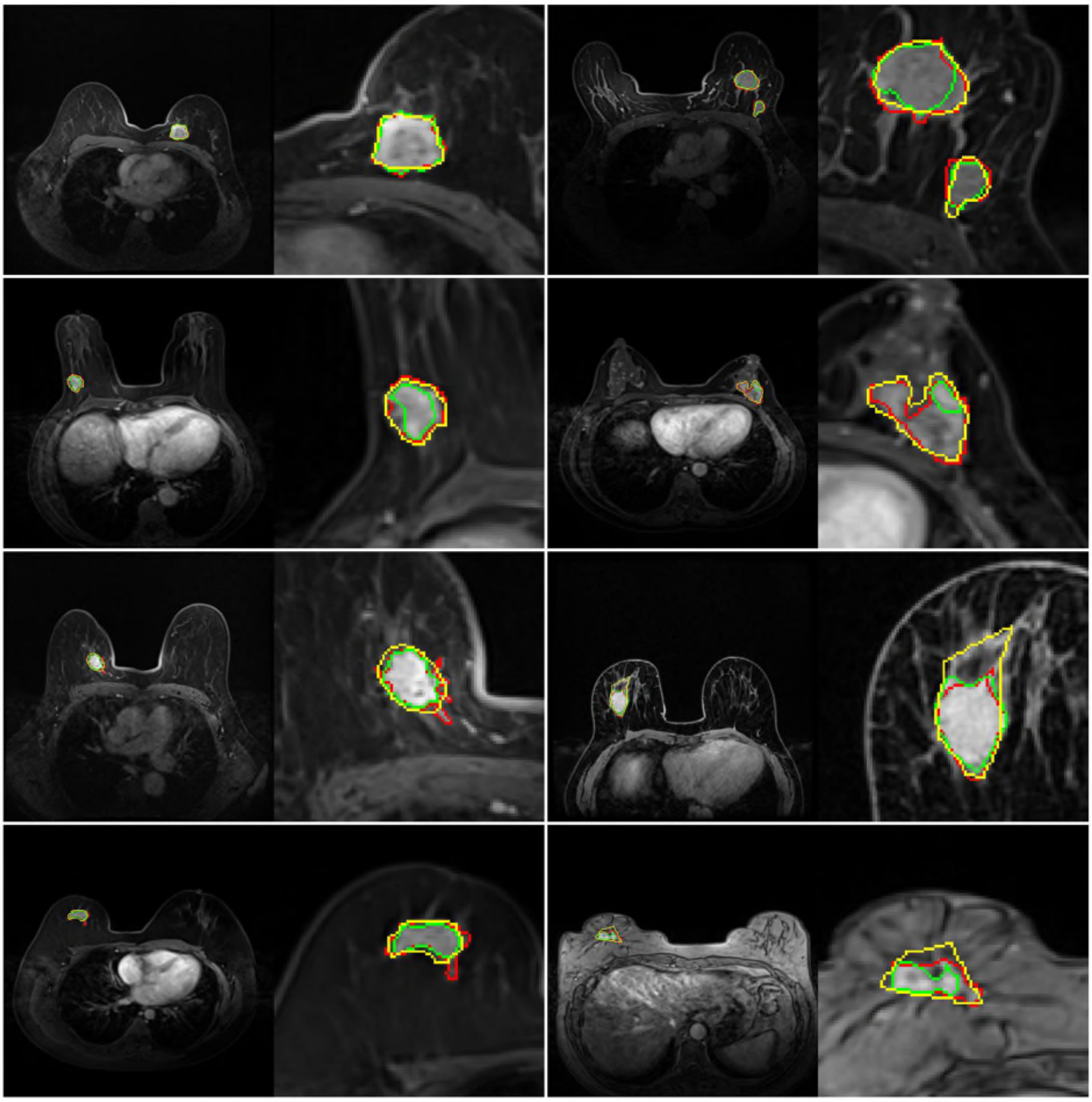

Supplementary Fig. 9 | Example breast tumor segmentation on the GPPH dataset. Red lines indicate the boundaries of the high-quality annotations. Green lines are the segmentation results of AIDE with 10% training annotations. Yellow lines refer to the segmentations obtained from an independent radiologist.

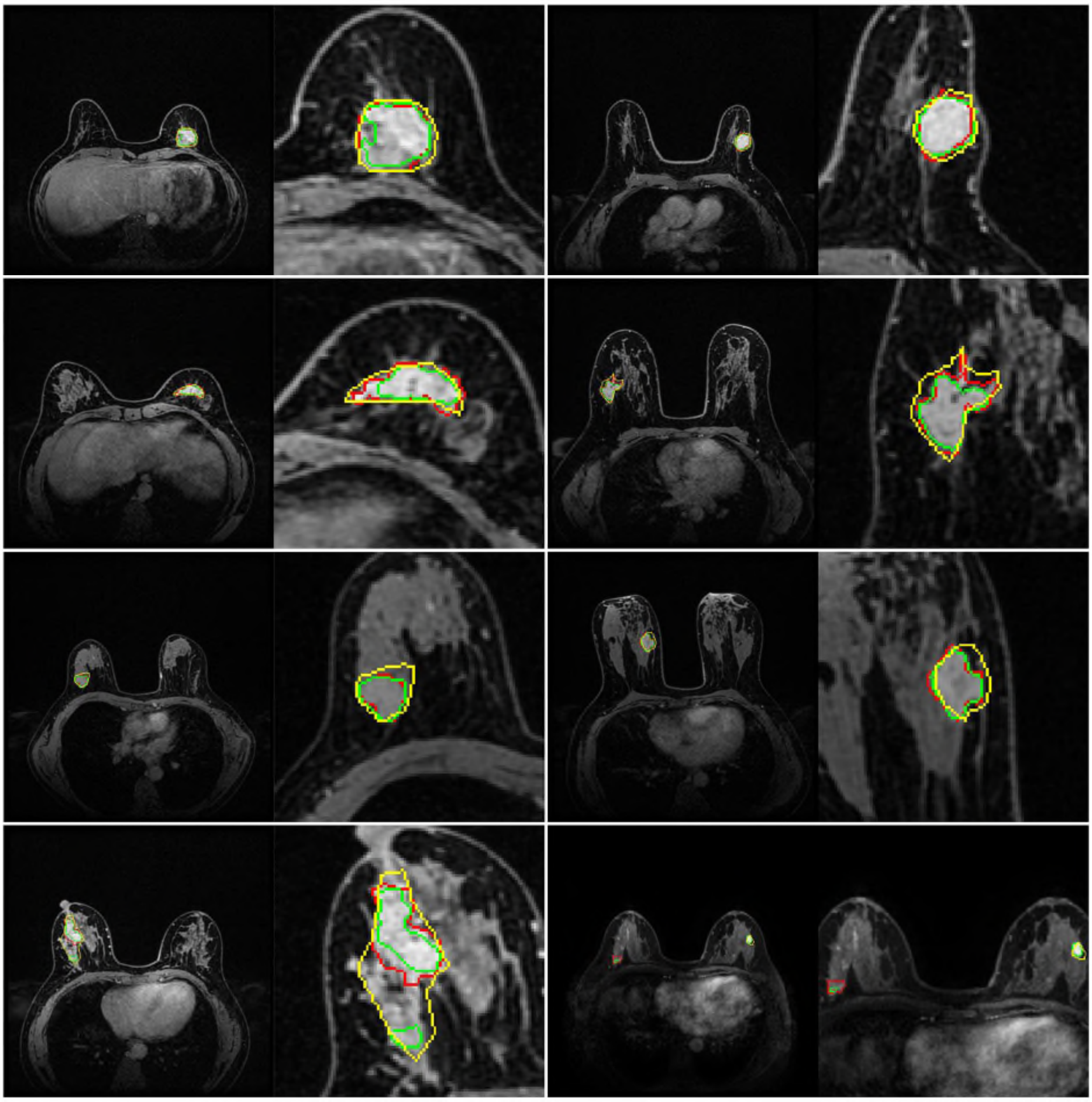

Supplementary Fig. 10 | Example breast tumor segmentation on the HPPH dataset. Red lines indicate the boundaries of the high-quality annotations. Green lines are the segmentation results of AIDE with 9.2% training annotations. Yellow lines refer to the segmentations obtained from an independent radiologist.

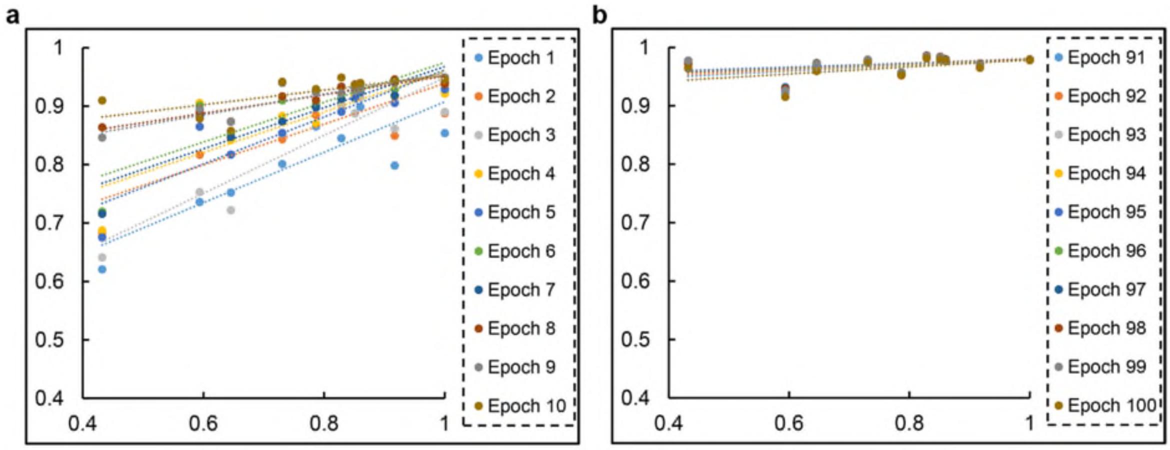

Supplementary Fig. 11 | Relationships between model memorization capability (DSCs calculated between network outputs and noisy labels (vertical axis)) and noisy label accuracy (DSCs calculated between noisy labels and high-quality labels (horizontal axis)) for the training set of the CHAOS dataset (training with 30 samples containing high-quality labels and 301 samples containing low-quality noisy labels utilizing the conventional fully-supervised learning method) in the first 10 epochs (**a**) and the last 10 epochs (**b**). Dots represent the DSCs calculated and dashed lines indicate the linear regression results.

Supplementary Table 1 | Segmentation results of networks under different SSL settings

Exp.	Noisy labels (%)	Train HQA	Train LQA	Total train	AIDE	DSC (%)	RAVD (%)	ASSD (mm)	MSSD (mm)
1	0	30	0	30	No	70.1	42.0	16.1	176.3
2	0	64	0	64	No	79.4	41.4	14.9	151.4
3	0	138	0	138	No	83.3	23.4	10.5	139.8
4	0	203	0	203	No	83.7	18.2	7.48	100.9
5	0	264	0	264	No	86.9	12.8	5.39	84.6
6	0	331	0	331	No	87.9	10.4	4.65	65.1
7	20.2	264	67	331	No	84.7	16.5	4.48	49.3
8	38.7	203	128	331	No	81.5	16.0	6.23	66.8
9	58.3	138	193	331	No	80.2	19.4	6.31	78.4
10	80.7	64	267	331	No	79.4	17.9	8.16	115.9
11	90.9	30	301	331	No	78.4	19.0	7.92	95.3
12	66.4	331	653	984	No	89.3	8.96	3.74	58.2
13	97.0	30	954	984	No	79.5	19.7	8.43	104.2
14	0	331	0	331	Yes	88.1	11.1	4.71	61.5
15	20.2	264	67	331	Yes	87.3	13.2	5.08	71.9
16	38.7	203	128	331	Yes	84.3	14.9	5.80	72.9
17	58.3	138	193	331	Yes	84.0	14.6	10.4	119.0
18	80.7	64	267	331	Yes	82.9	16.9	7.94	108.2
19	90.9	30	301	331	Yes	79.8	18.5	10.8	116.3
20	66.4	331	653	984	Yes	89.3	8.55	3.75	56.2
21	97.0	30	954	984	Yes	86.1	10.2	5.49	75.8

Supplementary Table 2 | Results of ablation studies on the CHAOS dataset

Temperature	Weight	Warm-up epoch	DSC (%)	RAVD (%)	ASSD (mm)	MSSD (mm)
0.5	1:10	20	86.5	10.0	4.55	48.8
2.0	1:10	20	86.5	10.1	4.55	48.6
1.0	1:1	20	86.3	11.5	4.49	48.1
1.0	1:20	20	86.8	9.71	4.15	43.9
1.0	1:10	10	86.1	11.8	4.49	45.1
1.0	1:10	30	86.7	11.3	4.29	46.3

Supplementary Table 3 | Segmentation results of networks trained and tested with prostate datasets of different domains

Training dataset	Testing dataset	AIDE	DSC (%)	RAVD (%)	ASSD (mm)	MSSD (mm)
Domain 1 labeled & 2 labeled	Domain 1	No	89.6	7.76	1.32	8.00
Domain 1 labeled & 2 labeled	Domain 2	No	89.0	9.19	1.21	7.27
Domain 1 labeled & 3 labeled	Domain 1	No	89.9	8.84	1.33	7.81
Domain 1 labeled & 3 labeled	Domain 3	No	85.7	9.55	1.74	10.3
Domain 2 labeled & 3 labeled	Domain 2	No	90.3	9.77	1.11	6.88
Domain 2 labeled & 3 labeled	Domain 3	No	87.0	7.91	1.51	9.43
Domain 1 labeled & 2 unlabeled	Domain 1	No	89.9	8.17	1.21	8.38
Domain 1 labeled & 2 unlabeled	Domain 2	No	33.7	71.5	5.94	20.68
Domain 1 unlabeled & 2 labeled	Domain 1	No	67.1	41.1	3.45	12.7
Domain 1 unlabeled & 2 labeled	Domain 2	No	83.1	20.7	1.68	9.70
Domain 1 labeled & 3 unlabeled	Domain 1	No	90.2	7.09	1.16	7.11
Domain 1 labeled & 3 unlabeled	Domain 3	No	44.8	66.6	5.65	18.4
Domain 1 unlabeled & 3 labeled	Domain 1	No	86.4	8.95	1.52	8.17
Domain 1 unlabeled & 3 labeled	Domain 3	No	81.7	8.98	2.44	15.7
Domain 2 labeled & 3 unlabeled	Domain 2	No	87.4	15.2	1.34	8.39
Domain 2 labeled & 3 unlabeled	Domain 3	No	54.6	56.7	4.95	18.1
Domain 2 unlabeled & 3 labeled	Domain 2	No	85.8	8.77	1.70	10.4
Domain 2 unlabeled & 3 labeled	Domain 3	No	86.9	9.83	1.47	7.80
Domain 1 labeled & 2 unlabeled	Domain 1	Yes	88.8	9.27	1.78	26.9
Domain 1 labeled & 2 unlabeled	Domain 2	Yes	80.0	25.1	2.83	18.2
Domain 1 unlabeled & 2 labeled	Domain 1	Yes	80.6	14.9	3.04	21.8
Domain 1 unlabeled & 2 labeled	Domain 2	Yes	86.0	14.4	1.68	11.4
Domain 1 labeled & 3 unlabeled	Domain 1	Yes	85.6	13.6	2.02	13.1
Domain 1 labeled & 3 unlabeled	Domain 3	Yes	74.0	40.4	3.56	17.8
Domain 1 unlabeled & 3 labeled	Domain 1	Yes	82.4	7.03	2.45	22.0
Domain 1 unlabeled & 3 labeled	Domain 3	Yes	84.2	9.71	1.69	9.42
Domain 2 labeled & 3 unlabeled	Domain 2	Yes	87.3	12.5	1.65	12.1
Domain 2 labeled & 3 unlabeled	Domain 3	Yes	74.5	38.6	3.77	20.3
Domain 2 unlabeled & 3 labeled	Domain 2	Yes	88.1	12.7	1.47	10.3
Domain 2 unlabeled & 3 labeled	Domain 3	Yes	85.9	8.54	1.57	8.67

Supplementary Table 4 | Segmentation results of methods for comparison for the unsupervised domain adaptation task

Source domain	Target domain	Pseudo -label	Co-teaching	DSC (%)	RAVD (%)	ASSD (mm)	MSSD (mm)
Domain 1	Domain 2	Y	N	53.9	41.5	4.39	17.1
Domain 1	Domain 3	Y	N	60.5	45.5	4.16	14.9
Domain 2	Domain 1	Y	N	70.5	32.4	3.33	14.8
Domain 2	Domain 3	Y	N	58.0	38.1	6.38	20.2
Domain 3	Domain 1	Y	N	82.3	18.0	2.02	10.2
Domain 3	Domain 2	Y	N	78.6	31.5	3.25	21.3
Domain 1	Domain 2	N	Y	58.1	49.2	4.28	17.0
Domain 1	Domain 3	N	Y	62.2	42.4	3.87	14.8
Domain 2	Domain 1	N	Y	74.1	26.9	2.87	14.2
Domain 2	Domain 3	N	Y	66.7	43.9	3.57	14.6
Domain 3	Domain 1	N	Y	81.9	8.88	2.43	15.8
Domain 3	Domain 2	N	Y	88.1	11.1	1.33	7.51
