# Supplementary material for: Annotation-efficient deep learning for automatic medical image segmentation
Source: Nat Commun. 2021 Oct 8;12:5915. doi: 10.1038/s41467-021-26216-9 (PMC8501087; doi:10.1038/s41467-021-26216-9)
Supplement: Supplementary file 2 — Reporting Summary [file 41467_2021_26216_MOESM2_ESM.pdf]

nature portfolio

Corresponding author(s): Hairong Zheng, Shanshan Wang, and Cheng Li

Last updated by author(s): Sep 21, 2021

Reporting Summary

Nature Portfolio wishes to improve the reproducibility of the work that we publish. This form provides structure for consistency and transparency in reporting. For further information on Nature Portfolio policies, see our Editorial Policies and the Editorial Policy Checklist.

Statistics

For all statistical analyses, confirm that the following items are present in the figure legend, table legend, main text, or Methods section.

n/a	Confirmed	
☐	☒	The exact sample size (n) for each experimental group/condition, given as a discrete number and unit of measurement
☐	☒	A statement on whether measurements were taken from distinct samples or whether the same sample was measured repeatedly
☐	☒	The statistical test(s) used AND whether they are one- or two-sided<br>*Only common tests should be described solely by name; describe more complex techniques in the Methods section.*
☒	☐	A description of all covariates tested
☒	☐	A description of any assumptions or corrections, such as tests of normality and adjustment for multiple comparisons
☐	☒	A full description of the statistical parameters including central tendency (e.g. means) or other basic estimates (e.g. regression coefficient) AND variation (e.g. standard deviation) or associated estimates of uncertainty (e.g. confidence intervals)
☐	☒	For null hypothesis testing, the test statistic (e.g. F, t, r) with confidence intervals, effect sizes, degrees of freedom and P value noted<br>*Give P values as exact values whenever suitable.*
☒	☐	For Bayesian analysis, information on the choice of priors and Markov chain Monte Carlo settings
☒	☐	For hierarchical and complex designs, identification of the appropriate level for tests and full reporting of outcomes
☒	☐	Estimates of effect sizes (e.g. Cohen's d, Pearson's r), indicating how they were calculated

Our web collection on statistics for biologists contains articles on many of the points above.

Software and code

Policy information about availability of computer code

Data collection	Public data were provided by the respective challenges. FDA-approved fully features PACS viewer (GE-Centricity version 5.0.0) was used to collect the breast image data.
Data analysis	The self-correcting algorithm package (v1.0.0) used to analyze the image data is available at https://github.com/lich0031/AIDE (DOI:10.5281/zenodo.5511736). The network implementation used the PyTorch deep learning framework (PyTorch 1.1.0). Result analyses are performed with custom code written in Python (python 3.5.6) with several packages, including numpy (numpy 1.15.2), pandas (pandas 0.23.4), PIL (Pillow 5.2.0), skimage (scikit-image 0.14.0), and SimpleITK (SimpleITK 1.2.0).

For manuscripts utilizing custom algorithms or software that are central to the research but not yet described in published literature, software must be made available to editors and reviewers. We strongly encourage code deposition in a community repository (e.g. GitHub). See the Nature Portfolio guidelines for submitting code & software for further information.

Data

Policy information about availability of data

All manuscripts must include a data availability statement. This statement should provide the following information, where applicable:

- Accession codes, unique identifiers, or web links for publicly available datasets
- A description of any restrictions on data availability
- For clinical datasets or third party data, please ensure that the statement adheres to our policy

The raw image data and relevant information of the utilized open datasets are accessible from the respective official websites of the challenges (CHAOS: https://chaos.grand-challenge.org/, NCI-ISBI 2013: https://wiki.cancerimagingarchive.net/display/Public/NCI-ISBI+2013+Challenge+-+Automated+Segmentation+of+Prostate+Structures, PROMISE12: https://promise12.grand-challenge.org/, and QUBIQ: https://qubiq.grand-challenge.org/) through standard procedures. The

clinical breast data were collected by the hospitals in de-identified format. Owing to patient-privacy considerations, they are not publicly available. All requests for academic use of in-house raw and analyzed data can be addressed to the corresponding authors. All requests will be promptly reviewed within 10 working days to determine whether the request is subject to any intellectual property or patient-confidentiality obligations, will be processed in concordance with intuitional and departmental guidelines, and will require a material transfer agreement (available at https://github.com/lich0031/AIDE).

Field-specific reporting

Please select the one below that is the best fit for your research. If you are not sure, read the appropriate sections before making your selection.

☒ Life sciences ☐ Behavioural & social sciences ☐ Ecological, evolutionary & environmental sciences

For a reference copy of the document with all sections, see nature.com/documents/nr-reporting-summary-flat.pdf

Life sciences study design

All studies must disclose on these points even when the disclosure is negative.

Sample size	No sample size calculation was performed. We used all samples available from the public and clinical datasets. Specifically, for the clinical datasets, three datasets with a total of 11,852 image samples acquired from 872 subjects by three medical centers were constructed. We randomly select 100 subjects as the test set for each dataset and the remaining as the training set. All the cases in the dataset were independent and non-repeating samples. The test sets were hold-out sets that were unseen by the models during model optimization. We believe the sample size was sufficient for our segmentation task to support our conclusions as there samples represent cases from different medical centers with different scanners and scan parameters.
Data exclusions	Before the research began, patients who had no visible breast tumors or underwent a biopsy, chemotherapy, or surgery before the MR image acquisition were excluded. The data exclusion criteria were pre-established. All the image data discussed in the methods section were included in the analyses.
Replication	The code used for training the deep-learning models are made publicly available for the reproducibility purpose. Statistical analysis has been given as well. Specifically, we run the code 3 times with different random initializations for the CHAOS dataset. For the domain adaptation task of prostate segmentation, 6 independent experiments were performed. On the QUBIQ datasets, we repeated 6, 7, 3, and 3 times respectively for the four different sub-tasks according to their dataset properties. For our breast datasets, data from three hospitals were utilized. So the experiments were performed independently for 3 times.
Randomization	The samples were allocated into experimental groups (training and testing) randomly for experiments utilizing the CHAOS dataset and the collected clinical breast datasets. For experiments utilizing the prostate datasets and the QUBIQ datasets, we allocated the samples following the respective challenges. According to the challenges, the samples were also randomly allocated into the different experimental groups.
Blinding	The radiologists were blinded to the group allocation during breast data collection and breast tumor delineation. The investigators in this study were not blinded to training and testing group allocation for the CHAOS dataset since the breast image data were de-identified before analyses and randomized group allocation was performed. For the prostate datasets and the QUIBIQ datasets, the investigators in this study were blinded to the group allocation.

Reporting for specific materials, systems and methods

We require information from authors about some types of materials, experimental systems and methods used in many studies. Here, indicate whether each material, system or method listed is relevant to your study. If you are not sure if a list item applies to your research, read the appropriate section before selecting a response.

Materials & experimental systems

n/a	Involved in the study	
☒	☐	Antibodies
☒	☐	Eukaryotic cell lines
☒	☐	Palaeontology and archaeology
☒	☐	Animals and other organisms
☐	☒	Human research participants
☒	☐	Clinical data
☒	☐	Dual use research of concern

Methods

n/a	Involved in the study	
☒	☐	ChIP-seq
☒	☐	Flow cytometry
☒	☐	MRI-based neuroimaging

Human research participants

Policy information about studies involving human research participants

Population characteristics	In total 872 female patients who had breast tumors were included in our study. These patients constitute three datasets from three hospitals. Dataset 1 consists of 300 patients with images collected between 25/04/2011 and 16/02/2017, and patients aged from 24 to 81 years (mean ± standard deviation: 50.05 ± 10.84 years). Dataset 2 consists of 200 patients with images collected between 25/10/2013 and 23/04/2019, and patients aged from 28 to 68 years (mean ± standard deviation: 46.85 ± 9.26 years). Dataset 3 consists of 372 patients with images collected between 05/01/2017 and 02/11/2018, and patients aged from 26 to 79 years (mean ± standard deviation: 48.23 ± 10.36 years).
Recruitment	The clinical data used for model optimization and testing were collected by the respective hospitals in a de-identified format. Retrospective data were aggregated and annotated by the hospitals. All MR scans were analyzed.
Ethics oversight	This retrospective study was approved by the Institutional Review Board of each participating hospital (Guangdong General Hospital, Guizhou Provincial People's Hospital, and Henan Provincial People's Hospital).

Note that full information on the approval of the study protocol must also be provided in the manuscript.
